# Efficacy of *Mycoplasma hyopneumoniae* vaccination before and at weaning against experimental challenge infection in pigs

**DOI:** 10.1186/s12917-016-0685-9

**Published:** 2016-03-29

**Authors:** Ioannis Arsenakis, Luca Panzavolta, Annelies Michiels, Rubén Del Pozo Sacristán, Filip Boyen, Freddy Haesebrouck, Dominiek Maes

**Affiliations:** Porcine Health Management Unit, Department of Reproduction, Obstetrics & Herd Health, Faculty of Veterinary Medicine, Ghent University, Salisburylaan 133, Merelbeke, 9820 Belgium; Department of Pathology, Bacteriology and Avian Diseases, Faculty of Veterinary Medicine, Ghent University, Salisburylaan 133, Merelbeke, 9820 Belgium

**Keywords:** Weaning, *Mycoplasma hyopneumoniae*, Vaccination, Efficacy, Strain

## Abstract

**Background:**

Commercial bacterins are widely used at weaning to control *Mycoplasma hyopneumoniae* infections in pigs. However, it is not known whether the efficacy of vaccinating against *M. hyopneumoniae* can be influenced by the weaning process when vaccination is applied at the day of weaning. The present study assessed the efficacy of a single *M. hyopneumoniae* vaccination (Ingelvac MycoFLEX®) three days before weaning (V1) or at weaning (V2) against experimental challenge infection. Four weeks after vaccination, groups V1 and V2 (*n* = 20 pigs each) and a non-vaccinated, positive control group (PCG) (*n* = 20) were endotracheally inoculated with a virulent *M. hyopneumoniae* field strain. Five pigs were used as a negative control group. All pigs were euthanized 5 weeks after challenge. The main parameters investigated included macroscopic and histopathological lung lesions at necropsy, immunofluorescence (IF) staining and quantitative real-time PCR (qPCR) on broncho-alveolar lavage (BAL) fluid for quantifying *M. hyopneumoniae*.

**Results:**

The average macroscopic lung lesion scores in groups V1, V2 and PCG were 0.54, 0.88 and 1.04, respectively (*P* > 0.05). The average lymphohistiocytic infiltration scores in groups V1, V2 and PCG were 2.95, 3.16 and 3.61, respectively (*P* < 0.05). The average IF scores were: V1 = 1.13, V2 = 1.19 and PCG = 1.25 (*P* > 0.05), the qPCR values were: V1 = 10^2.94^, V2 = 10^2.76^ and PCG = 10^3.23^ (*P* > 0.05). All pigs of the negative control group remained negative throughout the study.

**Conclusions:**

Both vaccinated groups had lower numbers of macroscopic and histopathological lung lesions, and lower numbers of *M. hyopneumoniae* organisms in the BAL fluid compared to the PCG. However, no firm conclusions could be made on whether weaning negatively influences the efficacy of *M. hyopneumoniae* vaccination, since significant differences between the treatment groups were only obtained for the histopathological lung lesions. This could be attributed to the fact that milder macroscopic lung lesions were produced in the inoculated pigs, when compared to previous trials conducted by the same group. Further research under field conditions is warranted to assess possible differences between the two vaccination strategies.

## Background

*Mycoplasma hyopneumoniae* is the etiological agent of enzootic pneumonia (EP), a chronic respiratory disease affecting mainly grow-finishing pigs [1, 2]. *M. hyopneumoniae* infections have been detected in almost all countries with intensive production systems and are responsible for major economic losses in the pig industry [1, 3]. These economic losses are due to pig growth retardation, higher feed conversion ratios, increased antimicrobial use and increased susceptibility to other respiratory pathogens such as *Pasteurella multocida*, *Actinobacillus pleuropneumoniae*, *Trueperella pyogenes* and *Streptococcus suis* [4]. *M. hyopneumoniae* is also an important pathogen involved in the porcine respiratory disease complex (PRDC) [5, 6].

Control of *M. hyopneumoniae* infections can be achieved in a number of ways, such as optimizing management and housing conditions, antimicrobial medication and vaccination. In many countries, more than 70 % of pig herds are vaccinated against *M. hyopneumoniae* in an effort to control the disease [7]. Vaccination with commercial bacterins has been extensively proven to reduce performance losses, the severity of clinical signs and lung lesions [8–12]. Different vaccination schemes can be implemented, depending on the type of herd, the production system, the infection pattern and the preference of the farmer.

Currently, both double and single vaccination strategies are widely practiced. Single vaccination is often administered either at 1 week of age or at weaning. Vaccination is often done at weaning because the piglets are handled then anyway, so vaccination can easily be included in routine farm management practices [12, 13]. On the other hand, weaning is one of the most stressful events in the pig’s life [14, 15]. The piglets are then experiencing the abrupt separation from the sow, they are being handled, they are being moved to other facilities, they are usually being mixed with other pigs, and they are receiving a different type of feed [14, 15]. Stress may interfere with an optimal response to vaccination, and therefore it is generally recommended not to vaccinate animals that are severely stressed [16].

Consequently, one important question that remains to be answered, and whose answer could contribute to the optimization of existing vaccination schemes, is whether the efficacy of vaccinating against *M. hyopneumoniae* can be influenced by the weaning process when vaccination is applied at the day of weaning. The objective of this trial was to investigate the efficacy of a one-shot vaccination applied either at weaning or three days before weaning in pigs experimentally infected with a virulent *M. hyopneumoniae* field strain.

## Results

### Clinical and performance parameters

In total, two pigs died during the trial, after receiving the endotracheal challenge infection (one in the V2 group and one in the PCG group). These pigs were not included in the analysis. In groups V1, V2 and PCG, coughing started approximately six days after challenge and continued to increase towards the end of the trial. In groups V1 and V2, coughing peaked between 66 and 70 days of age, whereas in the PCG, the peak was noticed at 80 days of age. The respiratory disease score (RDS) values for the period before challenge and the period after challenge, as well as for the overall period, are shown in Table 1. There were no significant differences between the groups (*P* > 0.05).

**Table 1:** Average (± standard deviation) of the daily RDS of the pigs for the three different periods

Age range (days)	V1 (*n* = 20)	V2 (*n* = 19)	PCG (*n* = 19)	*P* value
29–47	0.07 ± 0.26	0.28 ± 0.69	0.11 ± 0.37	0.108
48–83	0.79 ± 1.30	0.62 ± 1.20	0.65 ± 1.19	0.626
29–83	0.54 ± 0.94	0.50 ± 1.03	0.47 ± 0.91	0.852

Scores ranged from 0 (no coughing) to 6 (severe coughing undisturbed) (Halbur et al. [29]). Treatment groups: V1 (vaccinated before weaning, at 18 days of age), V2 (vaccinated on the day of weaning, at 21 days of age) and PCG (non-vaccinated). Overall and pairwise comparisons did not reveal any significant differences between groups for the three different periods. D48: experimental infection, D83: euthanasia

The average daily weight gain (ADG) results of groups V1, V2 and PCG during the period before challenge and during the period after challenge, as well as during the overall period, are presented in Table 2. There were no significant differences (*P* > 0.05) between the groups.

**Table 2:** ADG (± standard deviation SD) of each treatment group during the three different periods

	ADG (±SD) (g/pig/day)			
Age range (days)	V1 (*n* = 20)	V2 (*n* = 19)	PCG (*n* = 19)	*P* value
20–48	293 ± 41	299 ± 42	303 ± 55	0.491
48–83	701 ± 116	681 ± 129	718 ± 140	0.442
20–83	519 ± 56	511 ± 66	533 ± 71	0.461

Treatment groups: V1 (vaccinated before weaning, at 18 days of age, challenge infected), V2 (vaccinated on the day of weaning, at 21 days of age, challenge infected) and PCG (non-vaccinated, challenge infected). Overall and pairwise comparisons did not reveal any significant differences between the groups for the three different periods. D48: experimental infection, D83: euthanasia

### Macroscopic and histopathological lung lesions, and IF testing for *M. hyopneumoniae*

Only numerical differences in macroscopic lung lesions (*P* > 0.05) were found between groups V1, V2 and PCG (Table 3). The lowest average macroscopic lung lesion score was given to group V1 (0.54). The average lung lesion scores of the pigs in groups V2 and PCG were 0.88 and 1.04, respectively. There were no macroscopic lung lesions in the negative control group.

**Table 3:** Average (± standard deviation SD) values of macroscopic and histopathological lung lesion scores, IF scores, and qPCR

Parameter^a^	V1 (*n* = 20)	V2 (*n* = 19)	PCG (*n* = 19)	*P* value
Macroscopic lung lesions	0.54 ± 0.67^A^	0.88 ± 1.45^A^	1.04 ± 2.45^A^	0.777
Lymphohistiocytic infiltration	2.95 ± 0.50^A^	3.16 ± 0.58^B^	3.61 ± 0.59^C^	0.002
Percentage of air in lung tissue	44.28 ± 14.68^A^	37.47 ± 17.76^B^	31.46 ± 14.51^C^	0.000
IF	1.13 ± 0.54^A^	1.19 ± 0.79^A^	1.25 ± 0.70^A^	0.896
qPCR on BAL fluid	10^2.94^ ± 10^1.24A^	10^2.76^ ± 10^1.48A^	10^3.23^ ± 10^1.33A^	0.616

Treatment groups: V1 vaccinated before weaning, at 18 days of age, challenge infected, V2 vaccinated at weaning, at 21 days of age, challenge infected, and PCG positive control group i.e. non-vaccinated, challenge infected

Values with different superscript (A-C) in capital letter within a row are significantly different (*P* ≤ 0.05)

^a^ Macroscopic lung lesion scoring based on Hannan et al. [30]

Lymphohistiocytic infiltration score based on Morris et al. [31]

Percentage of air in lung tissue measured by means of automatic image analysis system (Optimas® 6.5, Media Cybernetics, Silver Spring, USA)

IF scoring based on Kobisch et al. [32]

Values from qPCR on BAL fluid expressed as copies of *M. hyopneumoniae*/mL

Statistically significant differences (*P* < 0.05) in histopathological lung lesions were present between groups V1, V2 and PCG (Table 3). The average lymphohistiocytic infiltration score was lower in group V1 (2.95) when compared with groups V2 (3.16) and PCG (3.61). The V1 group had the highest average percentage of air in the lung tissue (44.28), followed by group V2 (37.47) and PCG (31.46).

The results from the IF testing that was used to semi-quantitatively assess the load of *M. hyopneumoniae* organisms in the lung tissue, namely 1.13 (group V1), 1.19 (group V2) and 1.25 (PCG), were not significantly different (*P* > 0.05) between groups (Table 3). All pigs of the negative control group were negative for IF staining.

### qPCR assay and bacteriological examination

The number of *M. hyopneumoniae* organisms quantified by qPCR in the BAL fluid was not significantly different (*P* > 0.05) between groups V1, V2 and PCG (Table 3). Lower numbers of organisms were detected in groups V1 and V2 (10^2.94^ and 10^2.76^ copies/mL, respectively) compared to group PCG (10^3.23^ copies/mL). No *M. hyopneumoniae* DNA was detected in pigs of the negative control group.

Several bacteria were isolated from BAL fluid. In groups V1 and V2, *Bordetella bronchiseptica* (V1: 3/20 pigs; V2: 1/20 pigs) and polybacterial cultures (V1: 4/20 pigs; V2: 2/20 pigs) were obtained. In the PCG, *Haemophilus parasuis* (1/20 pigs), *S. suis* (1/20 pigs) and polybacterial cultures (1/20 pigs) were obtained. The bacteriological culture remained negative for all pigs in the negative control group.

### Serology

The serological results for *M. hyopneumoniae* at 21, 48 and 83 days of age are presented in Table 4. At 21 days of age, all the piglets were serologically negative for *M. hyopneumoniae*. At 48 days of age, 40 % of the pigs in each of the vaccinated groups V1 and V2 had seroconverted. None of the pigs in the PCG had seroconverted to *M. hyopneumoniae* at that time. At 83 days of age, all the pigs in groups V1 and V2 were seropositive, while 95 % of the pigs in the PCG had seroconverted. All pigs in the negative control group were serologically negative at all time-points.

**Table 4:** Percentage of seropositive pigs in the different groups at different time-points during the trial

Days of age	V1 (*n* = 20)	V2 (*n* = 19)	PCG (*n* = 19)
21^a^	0	0	0
48^b^	40	40	0
83^c^	100	100	95

Treatment groups: V1 (vaccinated before weaning, at 18 days of age, challenge infected), V2 (vaccinated on the day of weaning, at 21 days of age, challenge infected), PCG (non-vaccinated, challenge infected) and NCG (non-vaccinated, non-challenge infected)

^a^ Day of weaning and transport to the animal facilities of the Faculty of Veterinary Medicine, Ghent University

^b^ Inoculation time

^c^ Necropsy

## Discussion

The present experimental trial investigated whether the efficacy of one-shot vaccination is influenced by the weaning process. Differences between V1 (vaccination three days before weaning), V2 (vaccination at weaning) and the PCG (no vacination) were small and mostly statistically not significant, except for the microscopic lung lesions, which were lower in V1.

Apart from the transport related to the weaning process, the piglets in the present study were also transported to the experimental facilities, a trip that lasted anywhere from one-half to two hours. In fact, age- and site-segregated pork production is a complex process that involves movement of the piglets from the farrowing house to the nursery facilities, which are situated in different locations [17]. It can be expected, though, that the stress imposed on the piglets in our study may have been greater than when the piglets remain on the same site and are not transported (e.g. in single site farrow-to-finish pig herds) [18].

Vaccination at the moment of weaning, when the piglets are being handled anyway, is a common practice, as it can be implemented easily in the daily management of a pig herd. The pigs of V1 were vaccinated three days before weaning. This vaccination strategy is also commonly practiced by some pig producers in order to avoid any possible negative effects of the weaning process, and/or to avoid too much handling or interventions on the same day [19]. A difference of three days between the two vaccination groups most probably allowed sufficient time for the development of the first and most critical steps of the immune response prior to the stress of weaning [20]. At the same time, too great a difference in age at vaccination was avoided between the piglets in the V1 and V2 groups. In this sense, the two vaccination schemes are relevant for the situation in many pig herds.

The experimental infection model used for *M. hyopneumoniae* was similar to that used in previous trials [21–24]. Challenge infection proved to be successful since the vast majority of the infected pigs had positive qPCR values, exhibited the presence of *Mycoplasma*-like macroscopic lung lesions at necropsy and were positive for IF staining. However, the macroscopic lung lesions observed in the present trial were milder compared to trials that had been previously conducted using the same inoculation dose of *M. hyopneumoniae* F7.2C field strain, experimental facilities and way of allocation [23–25]. The precise reasons for this difference are not known. Based on the macroscopic lung lesion scores, serology, qPCR testing and IF staining, all the pigs in the negative control group were negative for *M. hyopneumoniae* throughout the trial, thus confirming that the study piglets were free of *M. hyopneumoniae*.

The RDS values in the PCG were similar to those mentioned in other experimental trials [22, 24]. Compared with the vaccinated groups V1 and V2, the only differences found in the PCG were numerical. There were no significant differences in ADG between the groups. This is mainly due to the high standard deviations observed and the limited numbers of pigs. ADG was measured, but it was not considered an important parameter for this experimental study. In vaccination trials under field conditions however, in which many more animals can be included, ADG is an important parameter.

Group V1 had the lowest numbers of macroscopic and histopathological lung lesions, and the lowest IF scores when compared to groups V2 and PCG. Nevertheless, significant differences between groups V1, V2 and PCG were only obtained for the histopathological lung lesions. This was confirmed by the results of the percentage of air parameter in the lung tissue measured by means of an automatic image analysis system. The statistically significant differences between groups V1, V2 and PCG proved that *M. hyopneumoniae* vaccination is effective in reducing lymphohistiocytic infiltration and increasing the percentage of air in the lung. This reduction of the lymphohistiocytic infiltration scores in the vaccinated groups is in accordance with previous experimental trials [22, 24]. The less severe histopathological lesions may be due to a modulation of the immune response in vaccinated animals. It has been shown that vaccination with a bacterin against *M. hyopneumoniae* reduces the infiltration of macrophages in the lung tissue [26].

Although lower qPCR values were recorded in the vaccinated groups when compared to the PCG, it was not possible to conclude whether vaccination was associated with the reduced bacterial load in the lungs, since the differences between the groups were only numerical. However, the detection of *M. hyopneumoniae* in the BAL fluid of groups V1 and V2 confirms that vaccination alone is not able to prevent colonization of the pig’s respiratory tract [4, 22, 24].

The pigs of groups V1, V2 and PCG were seronegative for *M. hyopneumoniae* on the day of transportation to the experimental facilities. At the time of inoculation, 40 % of the pigs in each of the vaccinated groups V1 and V2 had seroconverted. This is in agreement with other studies which have reported that vaccination with commercial bacterins induces seroconversion rates ranging from 30 to 100 % [4, 27]. At necropsy, all the pigs in groups V1 and V2 were seropositive, while the percentage of pigs that had seroconverted in group PCG was 95 %.

Overall, no firm conclusions could be made as to whether group V1 performed better than groups V2 and PCG, since only the microscopic lung lesions were significantly lower in the pigs in group V1. Further research under field conditions is necessary to elucidate the possible effect of weaning on the efficacy of one-shot *M. hyopneumoniae* vaccination. Field trials make it possible to include more animals and to study the entire course of *M. hyopneumoniae* infections up to the time of slaughter.

## Conclusions

In conclusion, the differences between the vaccinated groups V1 and V2 were small and mostly statistically not significant. Thus, no firm conclusions could be made as to whether the process of weaning had a negative impact on the efficacy of vaccinating against *M. hyopneumoniae*. A future field trial could possibly provide further insight on the same topic.

## Methods

### Animals

Sixty-five cross-bred piglets (Topigs 20 sows and Pietrain boars) were purchased from a herd that was free of *M. hyopneumoniae* and PRRSV. Sows and fattening pigs of different age groups in the herd had been repeatedly monitored clinically and serologically, and then by means of macroscopic pneumonia lung lesions at slaughter, and no evidence for the presence of *M. hyopneumoniae* or PRRSV in the herd was found at 3 weeks of age (day seven), the piglets were weaned and transported for one-half to two hours to the animal facilities of the Faculty of Veterinary Medicine, Ghent University, Belgium. From this point onwards, all the pigs received *ad libitum* a commercial, antibiotic-free feed.

### Experimental design and *M. hyopneumoniae* inoculation

At 14 days of age, prior to purchase, piglets from 20 different litters were individually ear-tagged and randomly allocated to four different groups. The three main treatment groups were: V1 (vaccinated before weaning, at 18 days of age and while still on the farm; *n* = 20), V2 (vaccinated during weaning, at 21 days of age and before being transported to the experimental facilities; *n* = 20) and a non-vaccinated positive control group (PCG; *n* = 20). Five pigs were used as a negative control group, to verify that they remained free of *M. hyopneumoniae*. They were not vaccinated. The pigs in groups V1 and V2 received a single-shot intramuscular (i.m.) injection (1 mL) of a commercial *M. hyopneumoniae* bacterin vaccine (Ingelvac MycoFLEX®, Boehringer Ingelheim Vetmedica). The pigs in PCG were left untreated and were not injected with a placebo to simulate field conditions. Upon arrival at the Faculty of Veterinary Medicine, each group was housed in a different experimental room equipped with a high efficiency particulate air filter to avoid possible transmission of the pathogen between groups.

At 48 days of age, the two vaccinated groups, V1 and V2, as well as the PCG were challenge infected endotracheally with a 7 mL inoculum containing 10^7^ color changing units per mL of the virulent *M. hyopneumoniae* F7.2C field strain as described previously [28]. The pigs in the negative control group were inoculated with 7 mL of sterile culture medium. For the inoculations, the pigs were anaesthetized by administering a mixture of Xyl-M® 2 % and Zoletil 100® i.m. at a dose rate of 0.3 mL/kg bodyweight.

All animals were euthanized and necropsied at 83 days of age. Deep anesthesia was induced by administering i.m. 0.3 mL/kg of a mixture of Xyl-M® 2 % and Zoletil 100®, followed by exsanguination. The study was approved by the ethical committee for animal experiments of the Faculty of Veterinary Medicine, Ghent University (EC2013/35).

### Parameters of comparison

#### Clinical and performance parameters

Each treatment group was examined daily for a minimum of 15 min for the presence of clinical signs throughout the entire trial period. Coughing severity was evaluated in a blinded manner by means of an RDS on a scale from 0 to 6 [29]. According to that scale: 0 (no coughing), 1 (mild coughing after being encouraged to move), 2 (mild coughing while at rest), 3 (moderate coughing after being encouraged to move), 4 (moderate coughing while at rest), 5 (severe coughing after being encouraged to move), and 6 (severe coughing while at rest). The daily RDS values were averaged for the three following periods: from day 29 until day 47, from day 48 (day of inoculation) until day 83 (day of necropsy), and from day 29 until day 83.

Individual bodyweights were measured at 20, 48 and 83 days of age to determine the ADG of each treatment group during the three following periods: from day 20 until day 48, from day 48 until day 83 and from day 20 until day 83.

#### Macroscopic and histopathological lung lesions, and IF testing for *M. hyopneumoniae*

After necropsy, *Mycoplasma*-like macroscopic lung lesions were quantified in a blinded manner using a lung lesion score diagram [30]. Theoretically, the total lung lesion scores could range between 0 (absence of lesions) and 35 (entire lung affected).

Two lung tissue samples per lobe (apical, cardiac and diaphragmatic) were collected from the right lung of each pig for histopathology and IF testing. Samples were collected from the edges of the lung lesions, if present [24].

For histopathology, the tissue was fixed in 10 % neutral buffered formalin and routinely processed and embedded in paraffin. One slide was made per lobe sample. Using light microscopy, the severity of peribronchiolar and perivascular lymphohistiocytic infiltration and nodule formation consistent with *M. hyopneumoniae* lesions were scored on a scale from 1 to 5 [31]. Scores 1 and 2 were classified as lesions not related to *Mycoplasma* infections. Scores 3, 4 and 5 were considered to be associated with *Mycoplasma* infections (mild, moderate and severe lesions characteristic of broncho-interstitial (cuffing) pneumonia, surrounding the bronchioles but extending to the interstitium, with lymphofollicular infiltration and mixed inflammatory cell exudates). After three lobes per pig were investigated, the average score per treatment group was calculated.

The percentage of lung area occupied by air (percentage air) was examined by means of an automatic image analysis system (Optimas® 6.5, Media Cybernetics, Silver Spring, USA). This percentage is inversely proportional to the severity of peribronchiolar and perivascular lymphohistiocytic infiltration in the lung tissue and the amount of intrabronchiolar – intrabronchial exudate. An average percentage per treatment group was calculated [28].

A direct IF assay was performed to assess the presence of *M. hyopneumoniae* in the lungs. The scores given could range from 0 to 3: 0 (no IF), 1 (limited IF), 2 (moderate IF), and 3 (intense IF) [32].

#### qPCR and bacteriological examination

During necropsy, BAL fluid was collected from the left part of the lungs before the tissue samples for histopathology and IF were taken [21]. The recovered fluid was divided into two aliquots, immediately cooled to 4 °C, and then stored at −70 °C until analysis. The first aliquot was used to quantify *M. hyopneumoniae* organisms by qPCR [33]. DNA was extracted (Qiagen, Blood and tissue kit, Belgium) and qPCR was performed using the CFX96 real-time PCR detection system (Bio-Rad). The analysis was done in a double-replicate single setting. To convert the threshold values to the number of organisms, a tenfold dilution series of *M. hyopneumoniae* DNA was used. Values below the last dilution were considered as negative.

The second aliquot of BAL fluid was used for routine bacteriological culture to detect the presence of *Actinobacillus* and *Haemophilus spp.*, and other pathogens which may affect the respiratory tract, such as *S. suis*, *B. bronchiseptica, P. multocida* and *T. pyogenes* [24].

#### Serology for *M. hyopneumoniae*

Blood samples were collected from all pigs at 21, 48 and 83 days of age to measure antibodies against *M. hyopneumoniae*, using a blocking ELISA (IDEIA, *M hyopneumoniae* EIA kit, Oxoid, UK) [12]. Sera with optical density (OD) values < 50 % of the OD_buffer-control_ were considered positive, while sera with OD values ≥ 50 % of the OD_buffer-control_ were considered negative.

### Statistical analysis

The statistical analysis was performed on groups V1, V2 and PCG. Pigs of the negative control group (*n* = 5) were not included, as they were only employed to ensure that the animals remained free of *M. hyopneumoniae* throughout the experiment. The RDS data were analyzed using repeated measures analysis of variance (ANOVA). Levene’s test was used to assess the homogeneity of the variances between the different groups. The ADG, macroscopic and histopathological lung lesions, percentage of air in the lungs, IF testing and qPCR were analyzed with a non-parametric Kruskal-Wallis ANOVA. These five continuous variables did not fulfill the criteria for homogeneity of variances. Statistical results were considered significant when the *P*-values were ≤ 0.05 (two-sided test). The statistical package SPSS 21.0 for Windows (SPSS 21, SPSS Inc., IL, USA) was used to analyze the data.
